# Bicycle helmet use and bicycling-related injury among young Canadians: an equity analysis

**DOI:** 10.1186/1475-9276-12-48

**Published:** 2013-07-02

**Authors:** Colleen M Davison, Michael Torunian, Patricia Walsh, Wendy Thompson, Steve McFaull, William Pickett

**Affiliations:** 1 Department of Public Health Sciences, Queen’s University, Kingston, Canada; 2 Kingston General Hospital, Kingston, Canada; 3 Department of Emergency Medicine, Queen’s University, Kingston, Canada; 4 Youth Policy, Division of Children, Seniors and Healthy Development, Public Health Agency of Canada, Ottawa, Canada; 5 Injury and Child Maltreatment Section, Health Surveillance and Epidemiology Division, Centre for Chronic Disease Prevention, Public Health Agency of Canada, Ottawa, Canada

## Abstract

**Introduction:**

Cycling is a major activity for adolescents in Canada and potential differences exist in bicycling-related risk and experience of injury by population subgroup. The overall aim of this study was to inform health equity interventions by profiling stratified analytic methods and identifying potential inequities associated with bicycle-related injury and the use of bicycle helmets among Canadian youth. The two objectives of this study were: (1) To examine national patterns in bicycle ridership and also bicycle helmet use among Canadian youth in a stratified analysis by potentially vulnerable population subgroups, and (2) To examine bicycling-related injury in the same population subgroups of Canadian youth in order to identify possible health inequities.

**Methods:**

Data for this study were obtained from the 6^th^ cycle (2009/10) of the Health Behaviour in School-aged Children (HBSC) study, which is a general health survey that was completed by 26,078 students in grades 6–10 from 436 Canadian schools. Based on survey responses, we determined point prevalence for bicycle ridership, bicycle helmet use and relative risks for bicycling-related injury.

**Results:**

Three quarters of all respondents were bicycle riders (n=19,410). Independent factors associated with bicycle ridership among students include being male, being a younger student, being more affluent, and being a resident of a small town. Among bicycle riders, 43% (95%CI ± 0.6%) reported never wearing and 32% (± 0.6%) inconsistently wearing a helmet. Only 26% (± 0.5%) of students reported always wearing a bicycle helmet. Helmets were less frequently used among older students and there were also important patterns by sex, geographic location and socioeconomic status. Adjusting for all other demographic characteristics, boys reported 2.02-fold increase (95% CI: 1.61 to 1.90) and new immigrants a 1.35-fold increase (95%CI: 1.00 to1.82) in the relative risk of bicycling-related injury in the past 12 months, as compared to girls and students born in Canada. The relative risk of injury did not vary significantly by levels of socioeconomic status.

**Conclusions:**

Troubling disparities exist in bicycle use, bicycle helmet use and bicycling-related injuries across specific population subgroups. Bicycle safety and injury prevention initiatives should be informed by disaggregated analyses and the context of bicycle-related health differences should be further examined.

## Introduction

The use of bicycles by children is a common and desirable activity in Canadian society. Several types of bicycle helmet legislation for young people have recently been adopted in various Canadian jurisdictions in an attempt to make bicycling a safer activity. In this paper, we examine bicycle helmet use and bicycling-related injury across population subgroups defined socially, economically and geographically in order to identify sub-population patterns and describe possible unfair and remediable differences, namely inequities that might exist and require attention.

There is a significant amount of existing literature related to the use and effectiveness of bicycle helmets and bicycle helmet legislation, including three relevant Cochrane Collaboration systematic reviews [1-3]. The literature is roughly divided into two types. First, there are studies that examine the relationship between helmets and injuries (see for example [1,2,4-9], and second, studies that examine helmet use itself including the many determinants of helmet use or non-use and the interventions associated with encouragement of the use of helmets (see for example [3,10-14]. There has been limited previous work that has examined the distribution of bicycling-related injury or helmet use. Bicycle and transportation safety education appears to be more successful with children under the age of 12 than with adolescents or young adults [15-17], there is some evidence suggesting the occurrence of sex and racial differences in helmet use [16,18] as well as evidence linking helmet nonuse and bicycling-related injury with lower socioeconomic status [17-23]. There remains a need for further research into group differences in bicycle helmet use and bicycle-related injury among young people to identify vulnerable population sub-groups beyond, and in addition to, those divided by sex, age and socioeconomic status and particularly youth in different social and geographic contexts.

The aim of our study was to identify sub-population patterns and possible inequities associated with bicycling, bicycle helmet use and bicycling-relating injury in young Canadians in order to better understand the health protection or health risk profile for these behaviours. Using data from Cycle 6 (2009/10) of the Canadian Health Behaviour in School-Aged Children Survey (HBSC), we addressed two study objectives: (1) To examine national patterns of bicycle and bicycle helmet use among Canadian youth by age, sex, school grade, socioeconomic status, number of years in Canada and urban–rural geographic location, and (2) To examine the point prevalence of bicycling-related injury and the association between these injury estimates and the same socio-demographic characteristics of Canadian youth.

## Methods

### Data source

The Health Behaviour in School-aged Children (HBSC) study is a cross-national survey conducted in collaboration with the World Health Organization. The 6^th^ cycle of the HBSC was undertaken in Canada in the 2009/10 school-year with 26,078 young Canadians, mostly aged 11 to 15, from 436 schools in 8 provinces and the 3 territories (all but Prince Edward Island and New Brunswick participated). The national sample was stratified by province/territory, type of school board (public vs. separate), urban–rural geographic status, school population size, and language of instruction (French vs. English) with standardized population weights generated to account for the over- or under-sampling in specific regions. Young people were not included in the survey if they were home schooled, attended private school, attended school on First Nations reserves, were incarcerated or did not provide informed consent (explicit or implicit, as per local school board protocol). The national study protocol was approved by the Queen’s University General Research Ethics Board. Response rates were 11/13 (84.6%) at the province/territorial level, 436/765 (57.0%) at the level of schools, and 26,078/33,868 (77.0%) at the individual student level.

### Variables for analysis

#### Bicycle and bicycle helmet Use

Question #86 of the survey asked: During the past 12 months, how often did you wear a helmet when you rode a bicycle? The response options were: I did not ride a bicycle; Never; Sometimes; Most of the time; and Always. Students who responded that they did not ride a bicycle were identified as non-riders. All other students were considered riders. Among riders, consistency in wearing a bike helmet (Never, Sometimes, Most of the time, and Always) was also reported. We collapsed the “sometimes” and “most of the time” categories into one category that we labeled *inconsistent* helmet use.

#### Youth Sub-groups

Demographic items were recorded for each student including sex (male/female), age (in years), school grade (<8, 8–9, 10+), years in Canada (lifetime/born in Canada, immigrant >5 years, immigrant ≤ 5 years); socio-economic status (self-perceived below average, average or above average) [24] and urban–rural status by population size of the students’ school’s census subdivision (rural = < 1000; small town = 1000–29,999; medium urban centre ≥ 30,000-99,999 and large urban centre ≥100,000). These urban–rural groupings are based on Statistics Canada suggested approach and cut-offs [25]. The grade categories roughly divide the students by younger (elementary), middle (equivalent to junior high school ages) and older (high school levels).

#### Injury

Experiences with bicycling-related injuries were derived from a question that asked whether participants had one or more injuries during the past 12 months that required treatment by a doctor or nurse. If injured, the students were then asked to think of their one most serious injury and then, what they were doing when the injury occurred. One of the response options to this follow-up question was bicycling/cycling. In this way we were able to identify a specific subset of bicycling-related injuries. Due to the lack of specificity of the available items, we were not able to specify further the exact type of bicycling-related injury that occurred.

### Approach to analysis

Bicycle ridership and bicycle helmet use were profiled using conventional descriptive statistics for population subgroups defined by age, sex, school grade, socioeconomic status, number of years in Canada and urban–rural geographic location. Poisson regression analyses, using the SAS Procedure PROC GLIMMIX which considered the weighted and multi-level (clustered) nature of the HBSC sampling design, were conducted to relate age, sex, urban–rural geographic location, socioeconomic status and number of years in Canada to reports of bicycle-related injury. Relative risks and associated 95% confidence intervals (with inflation for clustering) were estimated. All data analyses were conducted using SAS (version 9.3). We did not analyze injury by helmet use because the specific focus of this project was to profile bicycling and bicycling-related injury outcomes by different population subgroups, not to determine associations between injuries and use of helmets.

## Results

Of the overall sample of 26,078 students, 74% (95% CI ±0.53) of them reported riding a bike in the last 12 months. A description of the overall study sample and bicycle ridership by sex, age, school grade, socioeconomic status, urban–rural geographic location and number of years in Canada is provided in Table 1. Independent factors associated with bicycle ridership include being male, a younger student, more affluent, and from a small town.

**Table 1:** **A description of the overall study sample and the bicycle ridership **[1]

**Sub-group**	**Overall sample**	**Bicycle ridership**
	**n=26 078**	**n**^**1**^**=19 410 (row % 74.4)**
	**Sub-group totals in n (% of n)**	**Sub-group totals in n**^**1 **^**(% of n**^**1**^**)**
Sex:		
Boys	12 815 (49.2)	10 085 (52.0) (row % 78.7)
Girls	13 254 (50.8)	9322 (48.0) (row % 70.3)
Age:		
Mean (SD)	13.3 (1.6)	13.3 (1.6)
Range in years	9 to 19	10 to 19
Grade level:		
< 8	10 370 (39.8)	8180 (42.2) (row % 78.9)
8-9	10 661 (40.9)	7708 (39.8) (row % 72.3)
≥ 10	5047 (19.3)	3521 (18.1) (row % 69.8)
Socio-Economic status:		
Above average	13 998 (56.9)	10 817 (58.0) (row % 77.3)
Average	8276 (33.6)	6176 (33.1) (row % 74.6)
Below average	2339 (9.5)	1652 (8.9) (row % 70.6)
Urban–rural Location:		
Large Urban Centre	8589 (32.9)	6161 (31.7) (row % 71.7)
Medium Urban Centre	5739 (22.0)	4174 (21.5) (row % 72.7)
Small Town	10 767 (41.3)	8329 (42.9) (row % 77.4)
Rural	983 (3.8)	746 (3.8) (row % 75.9)
Years in Canada:		
Lifetime (born in Can.)	18 466 (71.5)	13 855 (72.0) (row % 75.0)
Immigrant > 5 yrs	6143 (23.8)	4642 (24.1) (row % 75.6)
Immigrant ≤ 5 yrs	1212 (4.7)	750 (3.9) (row 61.9)

^1^. Totals in each cell may not add exactly to corresponding “n” as the number of responses varies slightly by survey question.

Among the 19,410 students who reported that they rode bicycles, we examined patterns of bicycle helmet use. Overall, 43% (95%CI ±0.6%) of riders reported never wearing a helmet, 32% (95%CI ±0.6%) inconsistently wore a helmet and 26% (95%CI ±0.5%) reported always wearing a helmet while riding a bicycle in the past 12 months. Table 2 provides a breakdown of helmet use patterns by sex, age, school grade, socioeconomic status, urban–rural geographic location and years in Canada. Independent factors associated with always wearing a helmet include being a younger student, above average socioeconomically, and from a medium sized urban area.

**Table 2:** **Helmet use patterns among bicyclists by sub-group **[2]

**Sub-group**	**Never wears a helmet, (count and row %)**	**Inconsistently wears a helmet (count and row %)**	**Always wears a helmet, (count and row %)**
Sex:			
Boys (n= 10085)	4394 (43.6)	3102 (30.8)	2589 (25.7)
Girls (n= 9322)	3871 (41.5)	3019 (32.4)	2432 (26.1)
Age:			
Mean (SD)	13.9 (1.5)	13.1 (1.5)	12.5 (1.4)
Range in years	10 to 18	10 to 19	10 to 17
Grade level:			
< 8 (n= 8178)	2139 (26.2)	2852 (34.9)	3187 (39.0)
8-9 (n= 7708)	3794 (49.2)	2462 (32.0)	1452 (18.8)
≥ 10 (n= 3522)	2332 (66.2)	809 (23.0)	381 (10.8)
Socio-Economic status:			
Above average (n=10818)	4378 (40.5)	3501 (32.4)	2939 (27.2)
Average (n= 6176)	2785 (45.1)	1917 (31.0)	1474 (23.9)
Below average (n= 1653)	811 (49.1)	467 (28.3)	375 (22.7)
Urban–rural Location:			
Large Urban (n= 6161)	2597 (42.2)	1980 (32.1)	1584 (25.7)
Medium Urban (n= 4174)	1605 (38.5)	1345 (32.2)	1224 (29.3)
Small Town (n= 8329)	3714 (44.6)	2539 (30.5)	2076 (24.9)
Rural (n= 745)	349 (46.8)	257 (34.5)	139 (18.7)
Years in Canada:			
Born in Can. (n= 13,855)	5987 (43.2)	4255 (30.7)	3613 (26.1)
Immigrant > 5y (n= 4642)	1883 (40.6)	1544 (33.3)	1215 (26.2)
Immigrant ≤ 5y (n= 750)	331 (44.1)	275 (36.7)	144 (19.2)

^2^. Totals in each cell may not add exactly to corresponding “n” as the number of responses varies slightly for each survey question.

Among only the reported cyclists, we also examined the prevalence of bicycling-related injury and the association between the experience of an injury and a student’s demographic characteristics (age, sex, school grade, socioeconomic status, number of years in Canada and urban–rural geographic location). Percentages of students in each subgroup reporting a medically treated bicycle-related injury ranged from a high of 6.9% (among boys or new immigrant students) to a low of 3.4% among girls. Table 3 provides the crude and adjusted relative risk for bicycling-related injury for levels of the different demographic characteristics.

**Table 3:** Results of multiple logistic regression analysis examining direct effects of specific socio- demographic characteristics on risks for bicycling-related injury

**Socio-demographic characteristic**	**Crude relative risk**				**Adjusted relative risk**	
	**No.**	**% Injured**	**RR **[3]	**95% CI**	**RR **[4]	**95% CI**
**Sex**						
Female	310	3.4	1.00		1.00	
Male	671	6.9	2.13	[1.85-2.46]	**2.02**	**[1.78-2.30]**
**Age**						
≥ 15 yrs	429	6.4	1.00		1.00	
13-14 yrs	362	4.9	0.77	[0.66-0.90]	**0.77**	**[0.66-0.90]**
< 13 yrs	188	4.0	0.62	[0.53-0.78]	**0.62**	**[0.51-0.76]**
**Socioeconomic Status**						
Above Average	542	5.1	1.00			
Average	314	5.2	1.01	[0.88–1.16]		
Below Average	84	5.2	0.99	[0.79-1.25]		
**Urban–rural Geographic Location**						
Large urban	307	5.1	1.00			
Medium urban	189	4.7	1.01	[0.79-1.30]		
Small Town	442	5.5	1.10	[0.89-1.37]		
Rural	42	5.9	1.21	[0.79-1.85]		
**Years in Canada**						
Born in Canada	654	4.9	1.00		1.00	
Immigrant > 5 yrs	267	5.9	1.23	[1.06-1.42]	1.14	[0.98-1.32]
Immigrant ≤ 5 yrs	50	6.9	1.43	[1.07-1.93]	**1.35**	**[1.00-1.82]**

^3^. Estimated using multi-level procedures; students nested within schools, and SAS PROC GLIMMIX Procedure.

^4^. Model was adjusted for sex, age group, socio-economic status, urban–rural geographic status, and years in Canada. We have calculated adjusted relative risk estimates only for those variables that were included in the final regression model.

Adjusting for all other demographic characteristics, boys had a 2.02-fold increase (95% CI: 1.78-2.30) in the relative risk of bicycle-related injury relative to girls, while new immigrants a 1.35-fold increase (95%CI: 1.00-1.82) in the relative risk compared to those students born in Canada. Being younger was protective against bicycling-related injury. Although not statistically significant, students from small towns and rural settings showed slightly higher risk for bicycle-related injury relative to their peers from more urban settings. We found no statistically significant relationship between risk of bicycling-related injury and socioeconomic status.

## Discussion

We have estimated that about three-quarters of all young Canadians between the ages of 11 and 15 years ride bicycles. Given what is known about the potential risks for injury while cycling [7-21] and the protective effects of bicycle helmets [7,8,10-14] one would ideally see high rates of bicycle helmet use in all Canadian youth. Unfortunately, our estimates indicate that currently only about a quarter of young Canadian cyclists wear a bicycle helmet all of the time. Indeed, 43% of the cyclists in our study reported that they *never* wore a helmet. These aggregate trends are concerning, and when bicycle helmet uptake patterns are examined within specific socio-demographic groups of riders there also appear to be distinct, and potentially troubling, patterns. It seems quite clear that helmet use is less frequent among older students. Two-thirds of bicycle riders (66.2%) in grade ten or higher reported never wearing a helmet, compared to 26.2% in students grade 8 or lower. In many Canadian jurisdictions, there are helmet laws requiring use in younger riders and there has been a gradual shift towards increased use in the younger population over time [9,11,13]. There is also a social trend towards non-compliance as children age and take responsibility for their own health behaviours [15]. Helmet use patterns also appear to vary by sex, SES and geographic location indicating a possible association between the non-use of helmets and male sex, lower SES and more rural locations. We do encourage caution in the interpretation of these unadjusted point prevalence estimates however.

In addition to bicycle ridership and associated helmet use, the estimated relative risk of bicycling-related injury was also modeled for the 26,078 students in the study sample. Adjusting for all other factors, age, sex and immigration status were all independent predictors of the risk of injury. Indeed, new immigrants had a 1.35-fold increase in risk and male students a two-fold increase in risk for bicycling-related injuries than students in the comparison groups. While not strongly statistically significant, bicycling-related injury does appear to be more likely among youth in rural and small town areas as well. When taken in the context of existing literature, these findings are not entirely unexpected, but they do point to the importance of disaggregated analyses especially if research is to be used to informed public health intervention.

Health inequities are differences in health that are judged to be unfair and remediable [26-28]. Over the past twenty years, there has been a notable increase in attention and scholarship around health equity and inequity in Canada and globally. In the past five years, there have been many key publications such as “Closing the Gap in a Generation: Health equity through action on the social determinants of health” [29], Integrating Social Determinants of Health and Health Equity Into Canadian Public Health Practice [30]; Reducing Health Inequalities- A challenge for our times [31], the Chief Public Health Officer’s Report for Canada in 2008 [32] and Promoting Action on Equity Issues: A Knowledge-to-Action Handbook [33]. Striving for health equity means supporting all people to reach their full health potential and not be disadvantaged because of specific socio-demographic factors that influence their health and health behaviours. The fact that certain subgroups of populations are at greater apparent risks for bicycling-related injury is concerning, and particularly if these differences are unfair and remediable, they should be highlighted for attention and intervention. Canadian youth who are male, students who are older adolescents and students who are new immigrants to Canada are more at risk for bicycling-related injury. An aggregated analysis examining mean levels of risk, or overall health behaviours in young people as a homogenous group, would mask these differences. Of specific concern in this equity analysis are systemic factors that might exist as barriers to safe cycling for specific groups. While previous research indicates that older male students may, as a matter of choice, engage in higher risk activities that put them at greater risk for injury [16], this is not likely a similar pattern for new immigrant students. In this case, immigrant young people may be at greater risk as a result of transitions across different cycling environments and “safety cultures” for themselves and their families [34], or inadequate preparation or access to appropriate resources to support their safe cycling in Canada [35].

Previously, interventions to encourage bicycle helmets have tended towards whole population-level approaches including legislation, enforceable by law, requiring that all young people under the age of 18 years wear helmets. Two recent systematic reviews of bicycle helmet legislation for the reduction of bicycling-related head injury indicate that these approaches are useful [1,2]. There have also been non-legislative interventions aimed at the general public or broad groups of young people [3] and interventions to increase helmet uptake among lower income Canadians, for example [17,18,23]. In our review of the literature, we did not find examples of interventions catering specifically to male students or the older adolescent and young adult population specifically. These kinds of targeted interventions may be warranted. Also, from a policy perspective, there is a need to understand more about the mechanisms underpinning differences in bicycling-related outcomes so that interventions can be appropriately designed and evaluated. For example, are boys more likely to sustain bicycling related injuries because of the risk-taking behaviours they undertake that are different from girls? Is it just that they spend more time cycling than female youth? Or is it that they are somehow differentially impacted by environmental or policy factors? Have certain immigrant students been socialized within different bicycling cultures than Canadian-born students? If so, how do these possible differences influence their injury risk or their uptake of protective measures such as helmets? Are bicycling-related injuries more common in young people with older, less expensive bicycles or newer, perhaps more expensive ones? Answering these kinds of questions can inform targeted interventions to address root causes of potential inequities.

The most striking of the injury results from this study relates to the sex differences in estimated risk of bicycling-related injury (boys were 2.02 times more likely than girls to report an injury). Studies of gender equity, and the development of gender equity theory, tools and methodologies have a long history [36-38]. The Canadian government mandates sex and gender-based analysis (SGBA) and supports efforts across Ministries with policy frameworks and tools to address this form of inequity. This material could be more effectively engaged with in current public health and epidemiological research. For example, scholars of gender-based analyses have articulated the process of standardizing the assessment of health outcomes by gender [37] and this may be a helpful model for how disaggregation by various sub-groups could be realized more comprehensively across research and practice settings.

In the spring of 2012, the Ministry of Health and Long-term Care of Ontario updated their Health Equity Impact Assessment Tool (HEIA) [39]. This is a good example of a government tool and concerted initiative to support health across populations. The HEIA is underpinned by equity theory and health impact assessment methodologies [40-42]. The aim of the tool is to support health equity and reduce avoidable health disparities between population groups. What we find helpful about this tool is the explicit list of population sub-groups that might be considered in a health equity analysis. Table 4 is an adaptation of this list. While the sub-groups that are necessary to consider may differ on a case-by-case basis, further disseminating this kind of sub-population breakdown may better inform disaggregated analyses and support health equity research.

**Table 4:** Examples of population subgroups that can be considered in disaggregated analyses (Adapted from Ministry of Health and long-term care Ontario, Health Equity Impact Assessment tool, 2012)

**Sub-population group**	**Examples**
Aboriginal peoples	First Nations, Inuit, Métis
Age-related groups	Children, youth, seniors
Disability communities	Physical, deaf, deafened or hard of hearing, visual, intellectual/developmental, learning, mental illness, addictions/substance use, etc.)
Ethno-racial communities	Racial/racialized or cultural minorities, immigrants and refugees
Francophone or other linguistic groups	New immigrant francophones, deaf communities using sign language
Homeless	populations living on the street, marginally or under-housed
Low income	Unemployed, underemployed, single parents
Religious/faith communities	Muslim, Christian, Jewish
Populations as defined by geographic characteristics	Rural/remote, inner-urban, geographic or social isolation, under-serviced areas
Sex/gender	Male, female, women, men, trans, transsexual, transgendered, two-spirited
Sexual orientation	Heterosexual, lesbian, gay, bisexual
Other	Depending of the health issue or outcome of interest, other population subgroups may apply.

This study had a number of strengths. These included the survey and sample itself. The survey has been administered in four year cycles over 24 years in Canada (the 2009–2010 response rate was 75%) and it has been developed and honed by a strong and experienced international team (for a complete list of HBSC publications please see: http://www.hbsc.org/publications/). With an overall sample of more than 26,000 young people, sub-population group sizes remained sufficiently large to support analyses. Injury prevention and the health and health equity of children and youth clear priorities in Canada today and so the study has an important focus.

The study also has several limitations. As in other population health surveys, HBSC relies on accurate self-report data from children. Considerable efforts have been made over decades internationally to maximize validity and reliability of the survey items [24], however, some misclassification and non-response is inevitable especially for factors that are inherently difficult to quantify such as socioeconomic status. There is a possibility that non-responders varied significantly from responders and this could have biased our estimations of prevalence and, in reducing the size and heterogeneity of the sample, minimized our power to detect effects. In addition, we do not have any information about the frequency or context of bicycle usage or the specific types or frequency of bicycle-related injuries over the past 12 months. We also were not able to assess potential differences related to some of the HEIA population subgroups such as students with disabilities, students from different religious or faith communities, students from different racial or ethnic groups and students with different sexual orientations. Given our growing understanding of the social determinants of health equity [29,31,42], these kinds of analyses may have been helpful.

In summary, bicycling is a popular activity among Canadian youth and the health behaviours and possible risks associated with bicycling are not equally distributed across the Canadian youth population. More than 5% of young cyclists will experience a medically-treated bicycle-related injury each year in Canada. Injuries are more than twice as common among male students than female, and a third more common among new immigrants than non-immigrants. These differences are concerning. This study highlights the importance of considering disaggregated analyses when studying health behaviours, health outcomes and health equity in Canadian youth. These kinds of analyses can inform prevention interventions that, for bicycling-related injury for example, could be especially sensitive to gender, age, and socioeconomic differences. Beyond bicycles, there is evident value across health and health behavior topics, in analyzing and reporting data that are disaggregated by specific population subgroups. These kinds of analyses may help point to troubling disparities that may represent underlying, systemic inequities requiring targeted intervention and our continued public health efforts.

## Competing interests

The authors declare that they have no competing interests.

## Authors’ contributions

CD led the overall conduct of the study and preparation of the manuscript. MT, PW, WT, and SM represented the funders and contributed to the design of the study, the interpretation of findings and the review and editing of the final manuscript. WP led the analysis and made important contributions to the interpretation of findings and the writing of the final manuscript. All authors read and approved the final manuscript.
